# Isoflavones, Genistein and Daidzein, Regulate Mucosal Immune Response by Suppressing Dendritic Cell Function

**DOI:** 10.1371/journal.pone.0047979

**Published:** 2012-10-22

**Authors:** John Wei, Shiven Bhatt, Lisa M. Chang, Hugh A. Sampson, Madhan Masilamani

**Affiliations:** 1 The Jaffe Food Allergy Institute, Division of Allergy and Immunology, Department of Pediatrics, Mount Sinai School of Medicine, New York, New York, United States of America; 2 Immunology Institute, Mount Sinai School of Medicine, New York, New York, United States of America; Murdoch University, Australia

## Abstract

Lipopolysaccharide (LPS), a component of gram-negative bacterial cell walls, has been shown to have a strong adjuvant effect towards inhaled antigens contributing to airway inflammation. Isoflavones are anti-inflammatory molecules present in abundant quantities in soybeans. We investigated the effect of isoflavones on human dendritic cell (DC) activation via LPS stimulation and subsequent DC-mediated effector cell function both *in vitro* and in a mouse model of upper airway inflammation. Human monocyte-derived DCs (MDDC) were matured with LPS (or TNF-α) +/− isoflavones (genistein or daidzein). The surface expression levels of DC activation markers were analyzed by flow cytometry. Mature DCs +/− isoflavones were washed and cultured with freshly-isolated allogenic naïve CD4^+^ T cells for 5 days or with autologous natural killer (NK) cells for 2 hours. The percentages of proliferating IFN-γ^+^ CD4^+^ T cells and cytokine levels in culture supernatants were assessed. NK cell degranulation and DC cytotoxicity were measured by flow cytometry. Isoflavones significantly suppressed the activation-induced expression of DC maturation markers (CD83, CD80, CD86) and MHC class I but not MHC class II molecules *in vitro*. Isoflavone treatment inhibited the ability of LPS-DCs to induce IFN-γ in CD4^+^ T cells. NK cell degranulation and the percentage of dead DCs were significantly increased in isoflavone-treated DC-NK co-culture experiments. Dietary isoflavones suppressed the mucosal immune response to intra-nasal sensitization of mice to ovalbumin. Similar results were obtained when isoflavones were co-administered during sensitization. These results demonstrate that soybean isoflavones suppress immune sensitization by suppressing DC-maturation and its subsequent DC-mediated effector cell functions.

## Introduction

Exposure to airborne agents such as infectious microbes, chemicals, allergens and bacterial endotoxins has been linked to the development of airway inflammation and associated disorders such as airway hyper-responsiveness, asthma, COPD etc. Lipopolysaccharide (LPS), a component of gram-negative bacterial cell walls, exerts a strong Th1 adjuvant effect when combined with inhaled antigens, contributing to inflammation. However, low-level LPS stimulation has been shown to result in Th2-type immune responses [1]. LPS binds to toll like receptor-4 (TLR-4) and its co-receptor CD14 and LPS-binding protein [2]. Ligation of TLR-4 complex by LPS in antigen presenting cells (APCs) such as dendritic cells (DCs) initiates a signal transduction cascade involving MyD88, resulting in up-regulation of several genes associated with Th1 immunity [3], [4]. In addition, TLR-4 signaling induces a MyD88-independent, toll/IL-1 receptor -domain-containing adapter- inducing IFN-β (TRIF)-dependent pathway and resulting in interferon regulatory factor 3 activation and type I interferon synthesis [5], [6]. Whereas TRIF-signaling is also shown to be involved in maturation of dendritic cells [5], [7], [8], dual signaling of both MyD88 and TRIF was shown to be critical for maximal TLR4-induced DC maturation [9].

Both Th1 and Th2 cells have been shown to participate in antigen-specific airway inflammation and airway hyper-responsiveness in animal models [10], [11], [12]. Allergen challenge induced both Th1 and Th2-dependent genes in lungs in a murine model of asthma, however, Th1 genes were upregulated longer than Th2 genes [13]. Elevated levels of IL-4, IL-8, IFN-γ and IFN-γ-responsive STAT1 have been found in airways of severe asthmatic patients [14], [15], [16]. It has been well established that transfer of Th1 cells alone could trigger airway hyper-responsiveness in mice [11], [17], [18].

Several circumstantial and clinical pieces of evidence demonstrate the beneficial role of consumption of soybeans. Soybeans are the richest source of isoflavones such as genistein, daidzein and glycitein [19]. Other sources of isoflavones include red clovers [20] and *Psoralea corylifolia* plant family [21]. Isoflavones are a group of phytochemicals that are related to flavonoids. Isoflavones are well-known for their immunoregulatory and anti-inflammatory properties [22], [23], [24] and as inhibitors of protein kinase activity [25], [26]. Various *in vitro* and *in vivo* studies have documented their beneficial effects in human diseases such as cancer [27], [28], obesity [29], cardiovascular conditions, diabetic complications [30], Alzheimer's disease [31] and menopausal symptoms [32]. The consumption of a soy diet is directly correlated with decreased allergic rhinitis in the Japanese population [33] and a better lung function in patients with asthma [34]. Dietary soy supplementation reduces eosinophil leukotriene C4 synthesis and eosinophilic airway inflammation in asthmatic patients [35]. Isoflavones have been shown to inhibit asthmatic symptoms in guinea pig and mouse models of asthma [36], [37]. We have recently shown that dietary isoflavones suppress allergic reactions to peanut in a C3H/HeJ murine model [38].

There are several *in vitro* studies reporting the effect of isoflavones on different cell types and tissues [22]. However, how the individual cellular effects induce the overall immune response is still an active area of investigation. The exact mechanisms of action of isoflavones on target cells are yet to be fully understood. The direct effect of isoflavones on LPS adjuvanticity and the subsequent Th1 response has not been fully elucidated. In this report, we investigated the effect of isoflavones (genistein and daidzein) on DC activation via LPS/TLR-4 stimulation and subsequent DC-mediated effector cell function. We have tested two effector cell types that DCs could potentially interact with, namely T cells and natural killer (NK) cells. We further extended the study to investigate the effect of isoflavones on Th1 immune responses in a mouse model of upper airway inflammation.

## Materials and Methods

### Ethics Statement

Ethics approval for research involving laboratory animals was obtained from the Institutional Animal Care and Use Committee of the Mount Sinai School of Medicine (GCO Ref # 97-0452 #2) and has been performed according to the applicable guidelines. Human blood buffy coats from “anonymous donors” were purchased from New York Blood Center, New York and therefore exempt from obtaining ‘informed consent” and specific ethics approval from the institutional review board.

### Cells and reagents

Freshly-isolated peripheral blood mononuclear cells (PBMCs) were obtained from human buffy coats by Ficoll density centrifugation. PBMCs were washed, counted, and resuspended to 1×10^8^ cells/mL, and CD14^+^ monocytes cells were isolated by magnetic separation (StemCell Technologies). CD14^+^ monocytes were then cultured for 4 days with 10 ng/mL GM-CSF (R&D Systems) and 10 ng/mL IL-4 (R&D Systems) in RPMI 1640 medium (Life Technologies) supplemented with 10% FCS (Fisher Scientific), 2 mM glutamine, 100 U/mL pencillin and 100 µg/mL streptomycin (Life Technologies) to generate immature monocyte-derived DCs (MDDCs). Autologous NK cells were isolated from PBMCs by magnetic separation (EasySep™ human NK cell enrichment kit, StemCell Technologies). NK cells were cultured with 20 ng/mL IL-2 (R&D Systems) in RPMI 1640 medium supplemented with 10% FCS (Fisher Scientific), 2 mM glutamine, 100 U/mL pencillin and 100 µg/mL streptomycin. Naive CD4^+^ T cells were isolated from freshly-isolated PBMCs by magnetic separation (EasySep™ human naïve CD4+ T cell enrichment kit, StemCell Technologies). This kit includes an anti-CD45RO and other lineage specific antibodies. This methodology purifies naïve CD4^+^ T cells up to 95% purity. Therefore contamination of activated and memory T cells in the naïve CD4^+^ cells preparation is very minimal.

### DC maturation and co-cultures

MDDCs were matured with stimulants such as LPS (100 ng/mL), TNF-α (100 ng/mL) or CT (1 µg/mL) for 18 h in the presence or absence of 100 µM isoflavones (genistein or daidzein) along with vehicle dimethylsulfoxide (DMSO) controls. Following incubation and washing, cells were either stained (with antibodies against CD80 (B7.1), CD86 (B7.2), CD83, HLA-ABC, HLA-DR, HLA-E (BD Biosciences) and Live/Dead stain (Invitrogen) for flow cytometric analysis (BD-LSR2 flow cytometer, BD Biosciences and FlowJo software, Tree Star Inc.), or used for co-culture with NK or T cells. Cytokine levels were measured in culture supernatants by cytometric bead array (BD Biosciences).

2×10^5^ stimulant +/− isoflavone-treated DCs were washed and co-cultured with 8×10^5^ naïve CD4^+^ T cells (1∶4 ratio) in a mixed lymphocyte reaction (MLR) for five days. After five days, supernatants were tested for T cell cytokines. The cells were stained for T cell-specific antibodies, proliferation marker Ki-67 and Live/Dead stain. 1×10^5^ stimulant +/− isoflavone-treated DCs were washed and co-cultured with NK cells at a 1∶1 ratio for 2 hours. FITC-conjugated mouse anti-human CD107a (lysosome-associated membrane protein (LAMP)-1) and CD107b (LAMP)-2 antibodies (BD Biosciences) (2.5 µg/ml) were included in culture media. Following a 5-hour culture, supernatants were tested for cytokines. The cells were washed and stained with antibodies against CD56, HLA-DR, CD83, NKp46, and CD69 (BD Biosciences), and Live/Dead stain (Invitrogen) followed by flow cytometry. The expression of high levels of CD69 and CD107a/b in CD56^+^HLADR^lo^ NK cells indicated NK cell activation, and degranulation, respectively. DC death was measured with Live/Dead staining in CD83^+^HLADR^hi^ DCs.

### Murine airway inflammation model

Female Balb/c mice (5 per group) were fed a soy-free diet or a diet containing genistein and daidzein (1500 ppm each) for at least 2 weeks prior to the beginning of the sensitization protocol. Mice in each diet group were separated into two subgroups based on the following ovalbumin- Group I (50 µg OVA+ 5 µg LPS) and Group II (50 µg OVA + 5 µg LPS + 25 µg genistein + 25 µg daidzein). Mice were sensitized with the described quantities of OVA, LPS, and/or isoflavone in a final volume of 20 µl, 10 µl administered per nostril. Mice were sensitized once a week for 6 weeks. Blood samples were obtained via sub-mandibular bleeding at week 8. OVA-specific antibodies were estimated in serum by ELISA.

### Statistical Analysis

Statistical analyses were performed using GraphPad Prism software. Student t tests (two-tailed paired or unpaired with Welch's correction, as indicated) with confidence intervals of 95% were performed to obtain statistical significance (P value). P values ≤0.05 are considered significant.

## Results

### Isoflavones suppress LPS/TLR-4 signaling and DC maturation

**Figure 1:**
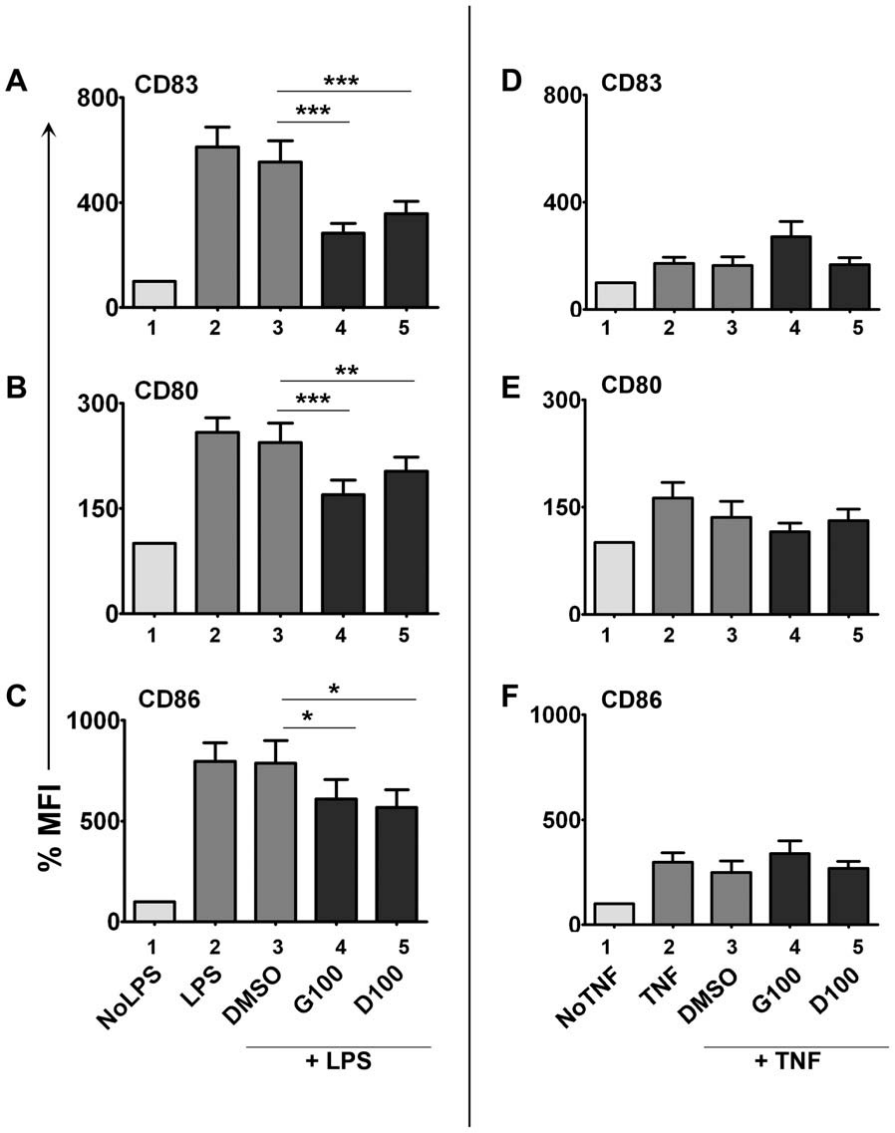
Suppression of LPS-induced DC marker expression by isoflavones. Human MDDCs were activated with 100 ng/mL LPS (A-C)- or 100 ng/mL TNF-α (D-F) +/− 100 µM genistein (G100) or daidzein (D100) for 18 h and stained with CD83 (A&D), CD80 (B&E) and CD86 (C&F). Isoflavone-treated cells are shown in dark bars (bars 4&5). DMSO - vehicle control for genistein and daidzein (bar 3). The percentage surface expression levels were calculated from the geometric mean fluorescent intensities where unstimulated controls (No LPS or No TNF, bar 1) were taken as 100%. The data pooled from at least 11 independent experiments from different cell donors for LPS stimulations and 7 experiments for TNF stimulations. At least two replicates were performed for each condition in every experiment. Statistical significance is indicated by ** (P≤0.01) or *** (P≤0.001) (paired student t-test).

Dendritic cells can be activated by various stimuli. During this process, DCs secrete several cytokines and up-regulate cell surface molecules required for the development of appropriate immune responses [39]. To investigate the specificity and the mechanistic role of isoflavones in DC activation, we generated human immature MDDC and activated them *in vitro* with different stimuli, such as LPS, IFN-γ or TNF-α in the presence or absence of isoflavones (genistein or daidzein 100 µM) for 18 h (Fig 1). The cells were washed, stained with fluorescent antibodies against CD83, CD80, and CD86 and analyzed by flow cytometry. The flow cytometric gating strategy and histograms of the staining are shown in Figures S1, S2 and S3. The percentage surface expression levels were compared to un-stimulated controls (No stimulant, taken as 100%) and shown as bar graphs. LPS treatment induced CD83 (Fig 1A), CD80 (Fig 1B), and CD86 (Fig 1C) expression on DCs' surface (mean fold increase compared to No LPS: 6.1, 2.6, and 8.0 respectively) –suggestive of DC maturation. The presence of genistein or daidzein during LPS treatment significantly inhibited the activation-induced up-regulation of these molecules when compared to LPS+vehicular control (DMSO). LPS-induced CD83 expression was reduced by 49% (P<0.001) and 36% (P<0.001) by genistein and daidzein respectively. Similarly, genistein and daidzein suppressed LPS-induced CD80 expression by 30% (P<0.001) and 15% (P<0.01) respectively. LPS-induced CD86 expression was suppressed by 22% (P<0.05) and 28% (P<0.05) respectively by genistein and daidzein. TNF-α also induced the expression of these molecules, albeit to a lesser degree than LPS. However, isoflavones did not have any significant effect on TNF-α-induced expression of these molecules (Fig 1D, -E & -F). The observed inhibition of LPS (but not TNF-α)-induced DC marker expression suggests a selective role of isoflavones in TLR-mediated DC activation and maturation. These results indicate that effects of isoflavones are specific to LPS-induced but not TNF-induced signal transduction pathways involved in DC activation. We also observed a dose dependency of isoflavones on LPS-induced expression of these molecules. CD83 in particular was maximally suppressed at a concentration of 100 µM isoflavones (Fig S4A & -B). Therefore, for subsequent experiments, we have used 100 µM concentrations of isoflavones. Isoflavones did not affect the cell viability at the concentrations used (Fig S4C).

### Isoflavones inhibit surface expression of MHC class I but not class II molecules in human MDDCs

**Figure 2:**
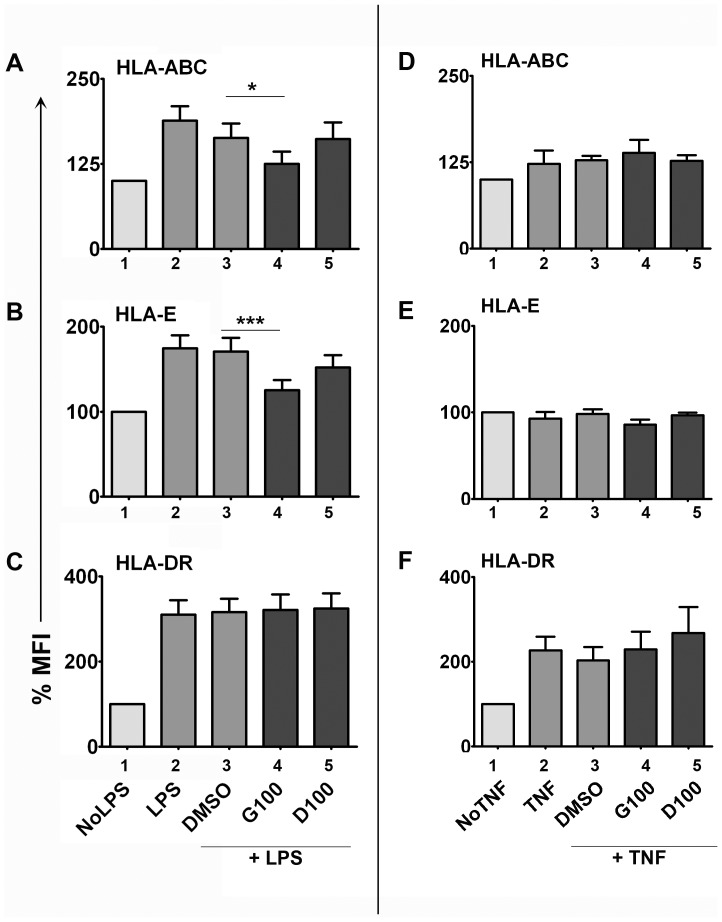
Effect of isoflavones on LPS-induced MHC expression on DCs. Human MDDCs were activated with 100 ng/mL LPS (A-C)- or 100 ng/mL TNF-α (D-F) +/− 100 µM genistein (G100) or daidzein (D100) for 18 h and stained with HLA-ABC (A&D), HLA-E (B&E) and HLA-DR (C&F). Isoflavone-treated cells are shown in dark bars (bars 4&5). DMSO - vehicle control for genistein and daidzein (bar 3). The percentage surface expression levels were calculated from the geometric mean fluorescent intensities where unstimulated controls (No LPS or No TNF, bar 1) were taken as 100%. The data pooled from at least 10 independent experiments from different cell donors for LPS stimulations and 5 experiments for TNF stimulations. At least two replicates were performed for each condition in every experiment. Statistical significance is indicated by ** (P≤0.01) or *** (P≤0.001) (paired student t-test).

MHC class I and class II molecules are expressed on DCs and also serve as markers of DC maturation. Therefore we tested the expression levels of MHC molecules on DCs activated with LPS +/− isoflavones. As expected, DCs upregulated both MHC class I (HLA-ABC & -E) (Fig 2A & B) and class II molecules (HLA-DR) (Fig 2C) upon activation with LPS (mean fold increase compared to No LPS: 1.9, 1.7, and 3.1 for HLA-ABC, -E and –DR respectively) –suggestive of DC maturation (as in Fig 1). The presence of isoflavones inhibited LPS-induced HLA-E by 28% (P<0.001) (Fig 2B). Similarly, LPS-induced HLA-ABC expression was significantly reduced by genistein by 34% (P<0.05). There was a non-significant trend towards decreased LPS-induced HLA-E and HLA-ABC by 13% and 15% by daidzein respectively (Fig 2A&B). Surprisingly, MHC class II (HLA-DR) expression was not affected by the presence of isoflavones during LPS-DC activation *in vitro* (Fig 2C). The failure to alter HLA-DR expression by isoflavones suggests that those cellular events that require HLA-DR (such as antigen presentation to Th cells) are not likely affected by isoflavones. This observation prompts us to hypothesize that isoflavone-mediated suppression of DC maturation is partial, allowing for more regulation, as opposed to a complete shut-down of DC maturation/function. For comparison, TNF-α and CT induced a modest increase in HLA-DR expression but not HLAs -A, -B, - C and –E (Fig 2 & Fig S5). Isoflavones did not affect the expression of MHC molecules in DC activation-induced by TNF-α or CT. It is likely that signaling pathways activated with TNF-α and CT do not participate in regulation of these molecules.

### Isoflavones inhibit cytokine secretion from LPS-activated DCs

**Figure 3:**
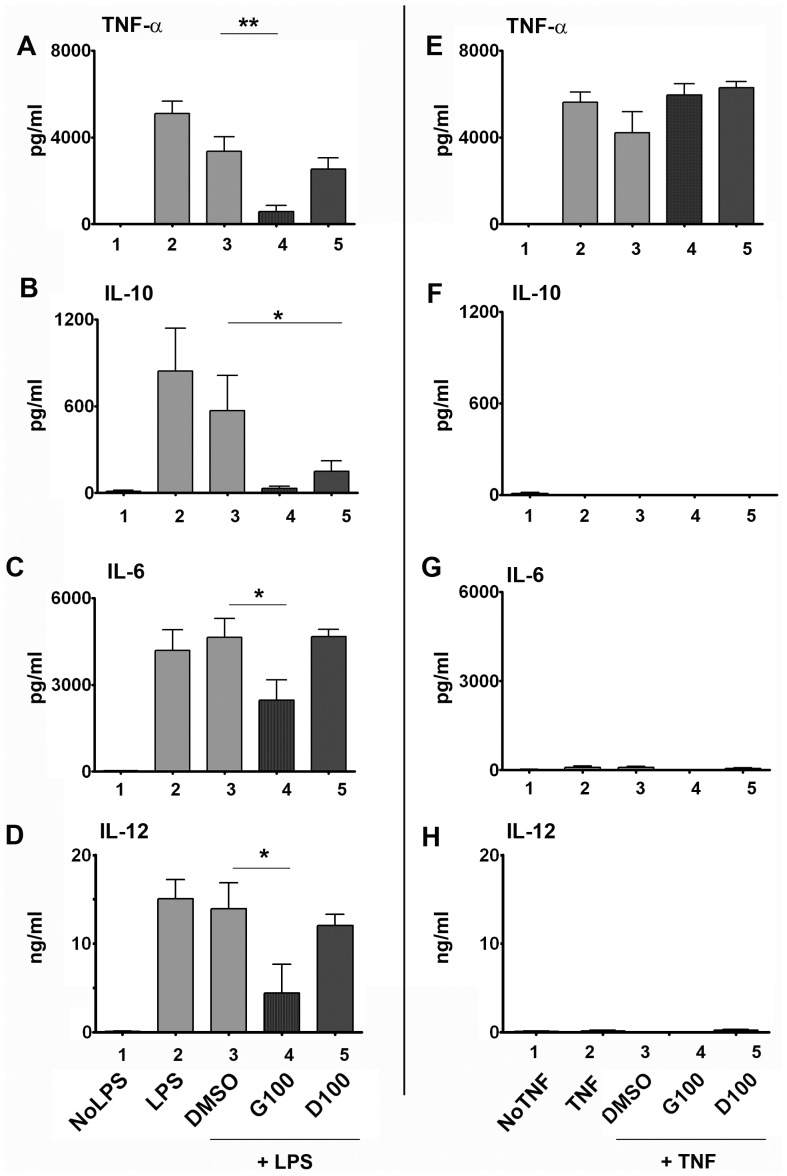
Isoflavones suppress LPS-induced cytokine secretion from DCs. Human MDDCs were activated with 100 ng/mL LPS or 100 ng/mL TNF-α +/− 100 µM genistein (G100) or daidzein (D100) for 18 h. TNF-α (A&E), IL-10 (B&F), IL-6 (C&G) and IL-12 (D&H) levels were measured in culture supernatants by cytometric bead arrays. Isoflavone-treated cells are shown in dark bars (bars 4&5). DMSO - vehicle control for genistein and daidzein (bar 3). The data pooled from at least 4 independent experiments from different cell donors for LPS stimulations and 4 experiments for TNF stimulations. Cytokine levels were measured in pooled culture supernatants from two replicate for each condition. Statistical significance is indicated by * (P≤0.05) (paired student t-test).

In addition to presenting antigens to T cells, DCs also secrete various cytokines that determine the outcome of T cell responses, i.e., Th1- or Th2-type immunity. We tested the cell culture supernatants of LPS-activated MDDCs +/− isoflavones for cytokines such as TNF-α (Fig 3A), IL-10 (Fig 3B), IL-6 (Fig 3C) and IL-12 (Fig 3D). LPS-induced DCs secreted substantial amounts of these cytokines. Genistein treatment suppressed LPS-induced TNF-α, IL-10, IL-6, and IL-12 secretion by 83% (P<0.01), 95% (P = 0.057), 55% (P<0.05) and 68% (P<0.05) respectively. While daidzein suppressed IL-10 secretion from LPS-treated DCs by 73% (P<0.05), secretion of other cytokines was not affected as much as genistein. Only TNF-α, but not IL-6, IL-10 and IL-12, was detectable in DC supernatants treated (and washed) with TNF-α +/− isoflavones (Fig 3E-H). Taken together with Figures 1 and 2, these data clearly demonstrate the specificity of isoflavones in modulating selective DC maturation pathways.

### Isoflavone-treated DCs regulate T cell activation and cytokine secretion

**Figure 4:**
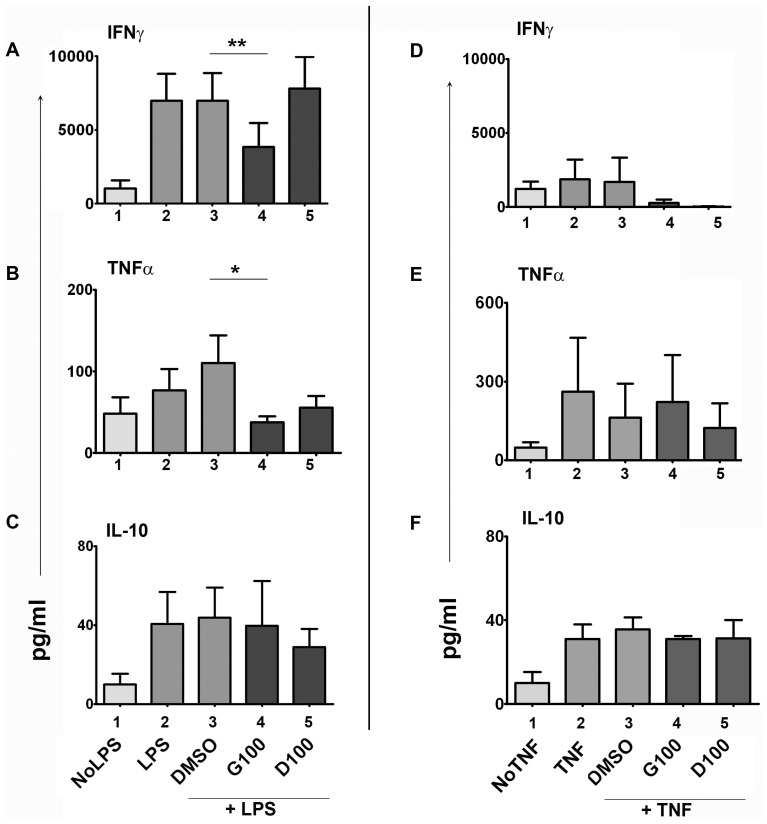
Isoflavones regulate DC-mediated T cell function. Human MDDCs were activated with 100 ng/mL LPS or 100 ng/mL TNF-α +/− 100 µM genistein (G100) or daidzein (D100) for 18 h, washed and incubated with allogenic CD4+ naïve T cells for 5 days. IFN-γ (A&D), TNF-α (B&E) and IL-10 (C&F) levels were measured by cytometric bead array in co-culture supernatants. Isoflavone-treated cells are shown in dark bars (bars 4&5). DMSO - vehicle control for genistein and daidzein (bar 3). The data pooled from at least 5 independent experiments from different cell donors for LPS stimulations and 2 experiments for TNF stimulations. Statistical significance is indicated by * (P≤0.05) (paired student t-test).

We tested the possibility that effective modulation of DC maturation pathways by isoflavones would be capable of regulating CD4^+^ T cell activation. Towards this end, we tested DC-induced T cell activation and maturation in a MLR. Equal numbers of MDDCs were activated with LPS or TNF-α +/− isoflavones as above, washed and cultured with allogenic naïve CD4^+^ T cells (1∶4 ratio) for 5 days. The DC / T cell co-culture cell supernatants were tested for IFN-γ, TNF-α and IL-10 secretion by cytometric bead arrays (Fig 4). LPS-activated DCs induced the secretion of these cytokines from CD4^+^ T cells (Fig 4A-C). IFN-γ (Fig 4A) and TNF-α (Fig 4B) but not IL-10 (Fig 4C) secretion were suppressed in LPS+genistein-DC / T cell co-culture supernatants (P<0.01, P<0.05 and P = 0.6 respectively). Only TNF-α (P = 0.1, non-significant trend), but not IFN-γ and IL-10 levels, were lower in LPS+daidzein-DC / T cell co-cultures. There were no significant differences in cytokine secretion in MLR cultures with TNF-α-DC / T cell co-cultures (Fig 4D-F). The co-cultured MLR cells were stained with the proliferation marker Ki67 along with other T cell and DC-specific antibodies. Isoflavone-treated DCs suppressed the proliferation of CD4^+^ T cells as indicated by reduced frequency of Ki67^+^ T cells in MLR cell cultures (Fig S6). These data suggest that DCs treated with isoflavones during the initial LPS maturation (but not TNF-α) affected the ability of DCs to induce MLR reaction and to induce cytokine production from activated CD4^+^ T cells. These data show the specificity of isoflavone action on regulating T cell activation.

### Regulation of NK cell function by isoflavone-treated DCs

**Figure 5:**
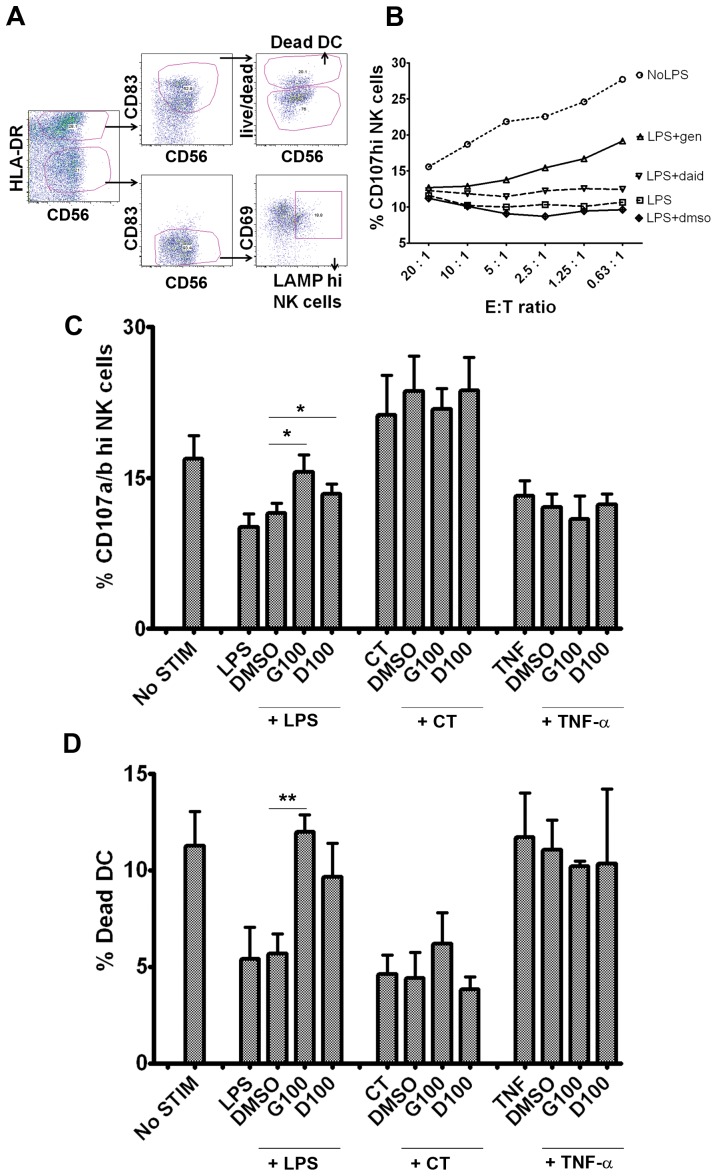
Isoflavones regulate DC-mediated NK cell degranulation and cytotoxicity. Flow cytometric gating strategy for DC-NK cell co-culture: Human MDDCs were activated with 100 ng/mL LPS, 1 µg/ml CT, or 100 ng/mL TNF-α +/− 100 µM genistein (G100) or daidzein (D100) for 18 h, washed and incubated with allogenic NK cells for 2 h in culture medium with FITC conjugated anti-human LAMP-1 and LAMP-2 Abs (2.5 µg/ml). The cells were then stained for CD83, CD56, CD69 and live/dead discriminator and analyzed by flow cytometry (A). Effector autologous NK cells were incubated with stimulant +/− isoflavone-treated target DCs (as above) in ratios ranging from 20∶1 to 0.63∶1 as indicated and stained as above. The percentage of LAMP-1/2^hi^ cells were plotted for each E:T ratio and stimulation condition as line graph (B). Effector allogenic NK cells were incubated with stimulant +/− isoflavones treated target DCs (as above) in 1∶1 ratio and stained as above (C & D). The percentage of LAMP-1/2^hi^ cells (C) and the percentage of DC cell death (D) are plotted for all the conditions and shown as bar graphs. DMSO - vehicle control for genistein and daidzein. The data pooled from 7 independent experiments from different cell donors for LPS stimulation, 5 experiments for CT stimulations and 2 experiments for TNF stimulations. DMSO : vehicle control. Statistical significance is indicated by * (P≤0.05) or ** (P≤0.01) (paired student t test).

As several MHC class I molecules also serve as ligands for NK cell receptors, the ability of isoflavones to down-regulate these molecules on DCs (Fig 2) seemed to suggest a potential role of isoflavones to influence DC-NK cell interactions. Therefore, we investigated this possibility through the use of DC-NK cell co-culture. MDDCs were generated and activated with LPS, TNF-α or CT +/− isoflavones as above, washed and cultured with autologous NK cells for 2 hours. Cell surface expression of the LAMP-1 and -2 (CD107a and CD107b respectively) was measured as a marker of cellular degranulation in activated, CD69^+^ NK cells and the percentage of cell death (using Live/Dead discriminator) in DCs as a measure of NK-mediated cytotoxicity (Fig 5A). We first performed NK-DC co-culture with varying effector (NK)-target (DC) (E:T) ratios ranging from 20∶1 to 0.6∶1 and stained for cell surface LAMP-1/2 (Fig 5B). There was a steady increase in NK cell degranulation (LAMP-1/2 staining) with decreasing E:T ratios (NK:DC). Optimal NK-DC interactions occurred when approximately equal numbers of NK cells and DCs were co-cultured. Co-culturing immature/unstimulated DCs with NK cells induced NK cell degranulation and cytotoxicity (Fig 5C & D). LPS treatment of DCs prior to co-culture with NK cells significantly inhibited NK cell activation and degranulation. In addition, LPS-matured DCs were also rescued from NK-mediated cytotoxicity (Fig 5C & D). These data correlate very well with the increased expression of MHC class I molecules on DC membranes upon LPS activation (Fig 2). Presumably, increased MHC class I interaction with inhibitory NK cell receptors negatively regulate NK cell function in this case. DCs matured with LPS in the presence of genistein or daidzein induced higher NK cell LAMP-1/2 expression (P<0.05 and P<0.05 respectively) and NK mediated cytotoxicity [P<0.01 and P = 0.06 (non-significant trend) respectively] when compared to DCs matured with LPS only. These data are suggestive of NK cell stimulation by immature DCs leading to increased NK cell degranulation (Fig 5C) and cytotoxicity (Fig 5D). Taken together with Fig 2 these results demonstrate that isoflavone-treated DCs are capable of activating NK cells and regulating the susceptibility of DCs to NK-mediated cytotoxicity through suppression of MHC class I molecules on the surface of DCs. DC activation by CT induces a signal transduction pathway (resulting in Th2 signals) in which only the expressions of CT-induced CD83 and CD80 are suppressed by isoflavones [38]. While CT treatment of DCs induced NK cell degranulation, we did not observe any significant effect of isoflavones on CT treatment (Fig 5C). Also, we did not observe high cell death in CT or CT+isoflavones treated DCs in NK-DC co-culture (Fig 5D). Similarly, there were no significant changes in NK cell degranulation or cytotoxicity in co-cultures with TNF-α treated DCs and NK cells (Fig 5C & -D). These results are in agreement with the finding that isoflavones have no effect on MHC expression in CT- or TNF-α-induced DCs (Fig S5). These data confirm that the CT and TNF signal transduction pathways are distinctively different from that of activation induced by LPS (Th1 signal) and that those signals are not affected by isoflavones. Moreover, only LPS-induced MHC class I expression is susceptible to isoflavone action, demonstrating the specificity of isoflavones.

### Isoflavones regulate mucosal immune response

We next examined our *in vitro* observations with an *in vivo* murine airway Th1 sensitization model. We investigated the effect of isoflavones on the humoral immune response towards OVA in Balb/c mice in two experimental conditions: 1) intra-nasal sensitization with OVA +/− isoflavones and 2) dietary administration of isoflavones followed by intra-nasal sensitization with OVA +/− isoflavones. Six- to eight-week-old female Balb/c mice were maintained on either a soy-free diet, or a diet containing 1500 ppm each of genistein and daidzein for the duration of the experiment. This concentration has been previously shown to result in plasma isoflavone levels roughly equivalent to that of levels seen in infants fed with soy formula [40]. Mice were further subcategorized into two groups, which were sensitized intra-nasally once weekly as follows: Group I (50 µg OVA+ 5 µg LPS), Group II (50 µg OVA + 5 µg LPS + 25 µg genistein + 25 µg daidzein). Mice were sensitized for three consecutive days on week 0 and then once a week for 6 weeks. Levels of anti-OVA antibodies were tested in serum by ELISA at week 8.

**Figure 6:**
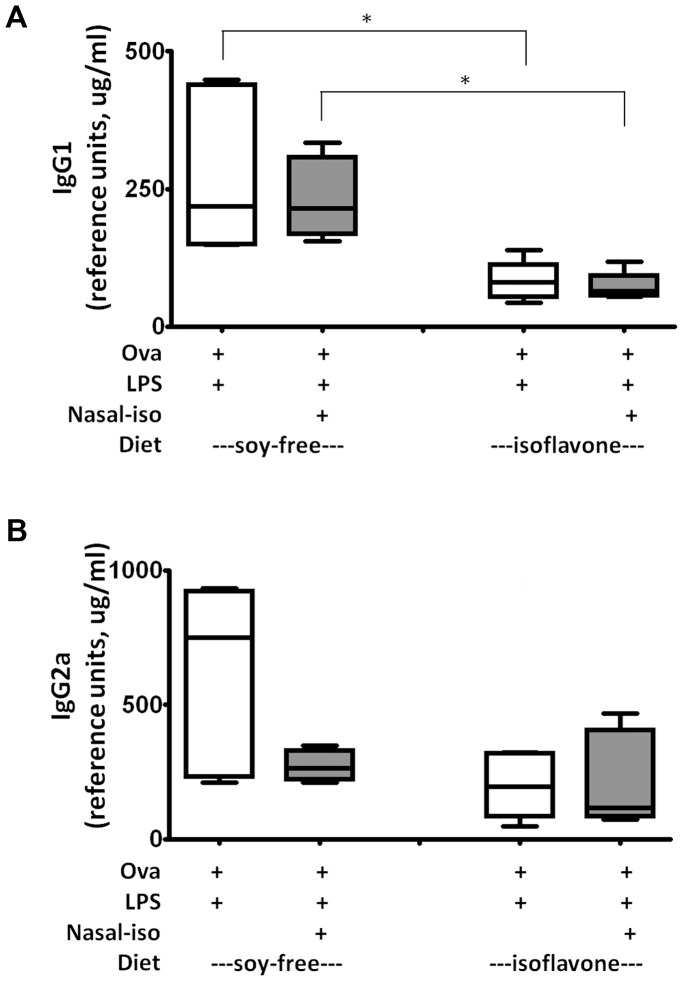
Effect of isoflavones on mucosal immune response. Balb/c mice (female, 5 per group) were fed either a soy-free or isoflavone-containing diet (Materials and Methods). Mice in each diet group were immunized with 50 µg OVA and 5 µg LPS as adjuvant +/− 25 µg isoflavones (genistein and daidzein). Serum samples were obtained after 8 weeks of sensitization and OVA-specific IgG1 (A) and IgG2a (B) levels were measured by ELISA. Statistical significance is indicated by * (P≤0.05) (Unpaired student t test with Welch's correction).

Mice fed a soy-free diet and sensitized intra-nasally with OVA along with LPS as adjuvant (Group I) induced OVA-specific IgG1 and IgG2a. In the group that received OVA+LPS+nasal isoflavones (Group II), we observed a trend towards lower OVA-specific antibody levels (Fig 6A &-B) compared to mice that received OVA+LPS (Group I) [mean IgG1 (µg/ml) = 230 Vs 279; mean IgG2a (µg/ml) = 272 Vs. 612; respectively]. These data demonstrate that the presence of isoflavones at the site of antigen sensitization tend to suppress the development of the immune response against the antigen, even over-riding the LPS adjuvant effects.

Interestingly, mice that received “dietary” isoflavones and were sensitized with OVA+LPS (+/− isoflavones) as above, showed an overall reduction in OVA-specific antibodies when compared to levels from similar group of mice fed a soy-free diet. The OVA+LPS group (Group I) that received “dietary” isoflavones showed lower OVA-specific IgG1 (P<0.05) and IgG2a (P = 0.07) when compared to a similar group of mice that received the soy-free diet (Fig 6 A & -B) [mean IgG1 (µg/ml) = 83; mean IgG2a (µg/ml) = 200]. Likewise, the OVA+LPS+isoflavone group (Group II) that received “dietary” isoflavones showed lower OVA-specific IgG1 (P<0.05) but not IgG2a (P = 0.55) when compared to a similar group of mice that received the soy-free diet (Fig 6 A & -B) [mean IgG1 (µg/ml) = 72; mean IgG2a (µg/ml) = 219].

In mice that received dietary isoflavones, the Ova-specific antibodies did not differ between groups I and II. These data suggest that dietary isoflavones have a more pronounced effect on the mucosal immune response than their direct action at the site of sensitization.

## Discussion

The majority of dietary isoflavones are present as glycosides. The bioavailability of dietary isoflavones is dependent on the extent of hydrolysis by the glycosidases of the intestinal microbiome and by the activity of epithelial lactase phlorizin hydrolase. The active aglycone forms of the isoflavones are immediately absorbed by epithelial cells and conjugated with glucuronides before entering circulation. Some of these molecules also enter the portal circulation and re-enter the digestive tract for second round of metabolism. Thus, the bioavailability of active aglycones for the cells and tissues other than the gut is comparatively low. However, the effect of circulating isoflavones could be pronounced in cells that express the enzyme beta-glucuronidases such as basophils, mast cells, eosinophils and most mucosal surfaces [41], [42], [43].

Isoflavones mimic the structure of estrogens and are weak agonists for the estrogen receptor. The phytoestrogenic property of isoflavones has prompted human safety concerns for soy food products, particularly in infants that receive soy milk as a substitute for cow's milk. However, the potency of isoflavone is about 1,000–0,000 times less than endogenous estrogens [44]. Moreover, dietary isoflavones from soy formula do not adversely affect human growth or reproductive development [45], [46]. To avoid the potential cross-reactivity with estrogens, animal studies involving isoflavone “injections” are performed in ovariectomized mouse models [24], which do not reflect physiological conditions *in vivo*.

As antigen-presenting cells, the ability of DCs to communicate with and activate other cells of the immune system is vital to generate an adaptive immune response. Antigen uptake by DCs initiates a complex maturation process and migration toward draining lymph nodes. During maturation, DCs up-regulate several co-stimulatory receptors that are involved in T cell activation such as members of the B7 family (CD80 and CD86), TNF super family (CD40) and MHC class II molecules [39]. In the lymph nodes, mature DCs act as APCs by loading the antigenic peptides onto MHC class II molecules to bind to the T cell receptor/CD3 complex. The MHC-TCR interaction provides the first signal to the T cell. A number of co-stimulatory and accessory molecules on DCs interact with their ligands on T cells, which provide a second signal, thereby activating T cells to proliferate, polarize and secrete a variety of cytokines [47]. Mature DCs can also present intact antigen to B cells [48] and can regulate NK cell function in the lymph nodes [49].

We have previously shown that dietary isoflavones (genistein and daidzein) suppressed allergic reactions to peanut in a C3H/HeJ mouse model and regulated CT-activated human MDDC function *in vitro* [38]. In this report, we extended this study to test the effect of isoflavones on Th1 immune response *in vitr*o and *in vivo*. We used LPS, a well-known Th1 skewing reagent to activate DCs and show that isoflavones regulate the maturation of DCs and DC-mediated effector functions *in vivo*. Isoflavones suppressed the expression of LPS-induced DC maturation markers, B7 co-stimulatory molecules and MHC molecules (Fig 1 & 2). Recently, Yum et al have shown that, daidzein suppressed LPS-induced DC co-stimulatory molecules including MHC class II molecules in mouse bone-marrow derived DCs [50]. However, in our case, genistein and daidzein did not affect the expression of HLA-DR in human MDDCs. Isoflavones selectively suppress the secretion of DC-derived cytokines (Fig 3). This regulatory mechanism in turn translates into reduced capacity of isoflavone-treated DCs to induce T cell activation as evident from a reduction of T cell proliferation (Fig S6) and cytokine secretion from T cells in a MLR culture (Fig 4).

There are *in vitro* studies that report conflicting results on the direct actin of isoflavones on NK cell function [51]. Here we report that NK cell function could be regulated indirectly by isoflavones through its effect on DCs. NK cells are found to be closely associated with DCs in the lymph nodes and NK-DC-T cell interactions are likely to take place in these environments [52], [53]. During NK cell-target cell interactions, NK cell function is regulated by a balance of signals from activating and inhibitory receptors [54]. These signals control the ability of NK cells to discriminate between normal and abnormal autologous cells. The major NK cell inhibitory receptors are CD94/NKG2A, killer cell immunoglobulin-like receptors (KIR) and immunoglobulin-like transcripts (ILT). CD4/NKG2A binds to the ubiquitously-expressed MHC class I molecule, HLA-E, (which presents a restricted set of peptides derived from other MHC class I leader sequences). The KIR and ILT family of proteins recognizes HLA-A, -B and –C molecules. The NK cell activation receptors represent a family of heterogeneous molecules, including NKG2D and members of “natural cytotoxicity receptors” such as NKp30, NKp44 and NKp46. Cognate interaction between NK cells and DCs results in cross-signaling [55], i.e., DCs can prime NK cell activation and proliferation, and as a result, NK cells can kill the target DCs, specifically the immature DCs, where the MHC class I expression is low [56]. This results in a NK-cell dependent DC editing process where appropriate mature DCs are selected for further antigen presentation and T cell priming [57]. It has been reported previously that DC-activated NK cells secrete IFN-γ and TNF-α [58]. Both immature and mature DCs can activate resting NK cells in the lymph nodes[56]. Activated NK cells then selectively kill immature DCs by DNAM-1 and NKp30 activation receptor ligation on NK cells [56], [59], [60]. Activated NK cells secrete high amounts of IFN-γ which is not only necessary for Th1 priming [61], but also for blocking the development of Th2 responses. Mature DCs also engage activation receptors on NK cells, but escape NK-mediated lysis because of high MHC class I expression on mature DCs, which provides strong inhibitory signals to NK cells thereby preventing cytotoxicity. In this light, our results (Fig 5) suggest that in the absence/reduction of MHC class I- mediated NK cell inhibition, DCs exposed to isoflavones are susceptible to NK cell-mediated killing. Thus, by reducing the expression of MHC class I expression on DCs, isoflavones contribute to regulation of NK cell function by activating NK cells and thereby enhancing NK cytotoxicity toward DCs and DC editing.

Our *in vitro* results are further verified in an *in vivo* nasal-sensitization mouse model. We tested the effects of local administration of isoflavone aglycones on Th1 sensitization, as well as the effect of conjugated-isoflavones in circulation (achieved through the diet). Balb/c mice were sensitized by nasal administration of OVA and LPS (as adjuvant), in the presence or absence of isoflavones. In addition, we performed an identical sensitization in mice fed with dietary genistein and daidzein (Fig 6). We show that isoflavones (both dietary and at the site of sensitization) suppress the induction of immune response against the mucosal antigen, OVA. However, the effect of “dietary isoflavones” on the mucosal immune response is more pronounced than the local effect of isoflavones at the site of sensitization. This is evident by significantly lower OVA-specific antibody levels in mice that received isoflavone diet than the soy-free diet. In our mouse model, the presence of isoflavones at the site of sensitization could suppress the antigen uptake, maturation and antigen presentation function of mucosal DCs there by suppressing the initiation of immune response and antibody production. It is also likely that the epithelial cell functions are affected during antigen exposure, as dietary isoflavones also suppress the immune response against ovalbumin. When isoflavones are administered in the diet, the overall immune response toward OVA was suppressed including the adjuvant effect of LPS. Additional administration of isoflavones at the site of sensitization along with the antigen in mice fed an isoflavone-containing diet was not required for the suppression of the immune response. The possible explanations for this observation is that dietary isoflavones are absorbed in conjugate forms and converted into active aglycones at mucosal surfaces, which in turn suppress mucosal immune responses.

In conclusion, our data clearly demonstrate the immuno-regulatory role of isoflavones. These results could have wide implications in future treatment strategies of respiratory hypersensitivities and allergies.

## Supporting Information

Figure S1 MDDC flow cytometry. The expression levels of CD80, CD83, CD86 and HLA-DR were tested in FSC^hi^ “live” MDDCs treated with LPS+/−genistein or daidzein. A representative staining is shown as dot plots (A-D) and histogram overlays (E&F).
(TIF)

Figure S2 MDDC flow cytometry. The expression levels of HLA-ABC and HLA-E were tested in FSC^hi^ “live” MDDCs treated with LPS+/−genistein or daidzein. A representative staining is shown as dot plots (A-D) and histogram overlays (E).
(TIF)

Figure S3 MDDC flow cytometry. The expression levels of CD80, CD83, CD86, HLA-ABC HLA-E and HLA-DR were tested in FSC^hi^ “live” MDDCs treated with LPS+/−genistein or daidzein. A representative staining is shown as dot plots (A-D) and histogram overlays (E&F).
(TIF)

Figure S4 Isoflavone titration. MDDCs were activated with 100 ng/mL LPS in the presence or absence of titrated amounts of genistein (A) or daidzein (B). The concentration of isoflavones ranged from 3 µM to 100 µM. The percentage surface expression levels of CD83 were calculated from the geometric mean fluorescent intensities where unstimulated control (No LPS) was taken as 100% and shown as bar graphs. Vehicle control (DMSO, µL) for conditions from 12.5 to 100 µM are shown. Flow cytometric assessment of cell viability using live/dead stain (Invitrogen) was performed in cells treated LPS +/− isoflavones (100 µM) (C). No difference in cell death was found in all the conditions employed.
(TIF)

Figure S5 Effect of isoflavones on CT-induced MHC expression on DCs. MDDCs were activated with 1 µg/mL CT for 18 h and stained with HLA-ABC (A), HLA-E (B) and HLA-DR (C). DMSO - vehicle control for genistein and daidzein. The percentage surface expression levels were calculated from the geometric mean fluorescent intensities (No LPS or No TNF controls taken as 100%) and shown as bar graphs. The data pooled from at least 4 independent experiments from different cell donors. At least two replicates were performed for each condition in every experiment. There was no significance in the expression levels of these molecules between the conditions employed.
(TIF)

Figure S6 Isoflavones regulate DC-induced T cell proliferation. MDDCs from two donors were activated with 100 ng/ml LPS +/− 100 µM genistein (G100) or daidzein (D100) for 18 h and washed. Each donor DCs were incubated with two allogenic CD4^+^ naïve T cells from two other different donors for 5 days. The cells were stained with fluorescent-conjugated antibodies against CD3, CD83 and Ki67 along with live/dead discriminator dye. The frequency of CD3^+^CD83^−^ cells in Ki67+ gate in No stimulation (No LPS) condition was taken as 100% and the fold change in Ki67^+^ T cells in LPS+dmso, LPS+genistein and LPS+daidzein cultures were plotted.
(TIF)
