# Supplementary figures and images for: Isoflavones, Genistein and Daidzein, Regulate Mucosal Immune Response by Suppressing Dendritic Cell Function
Source: PLoS One. 2012 Oct 22;7(10):e47979. doi: 10.1371/journal.pone.0047979 (PMC3478285; doi:10.1371/journal.pone.0047979)

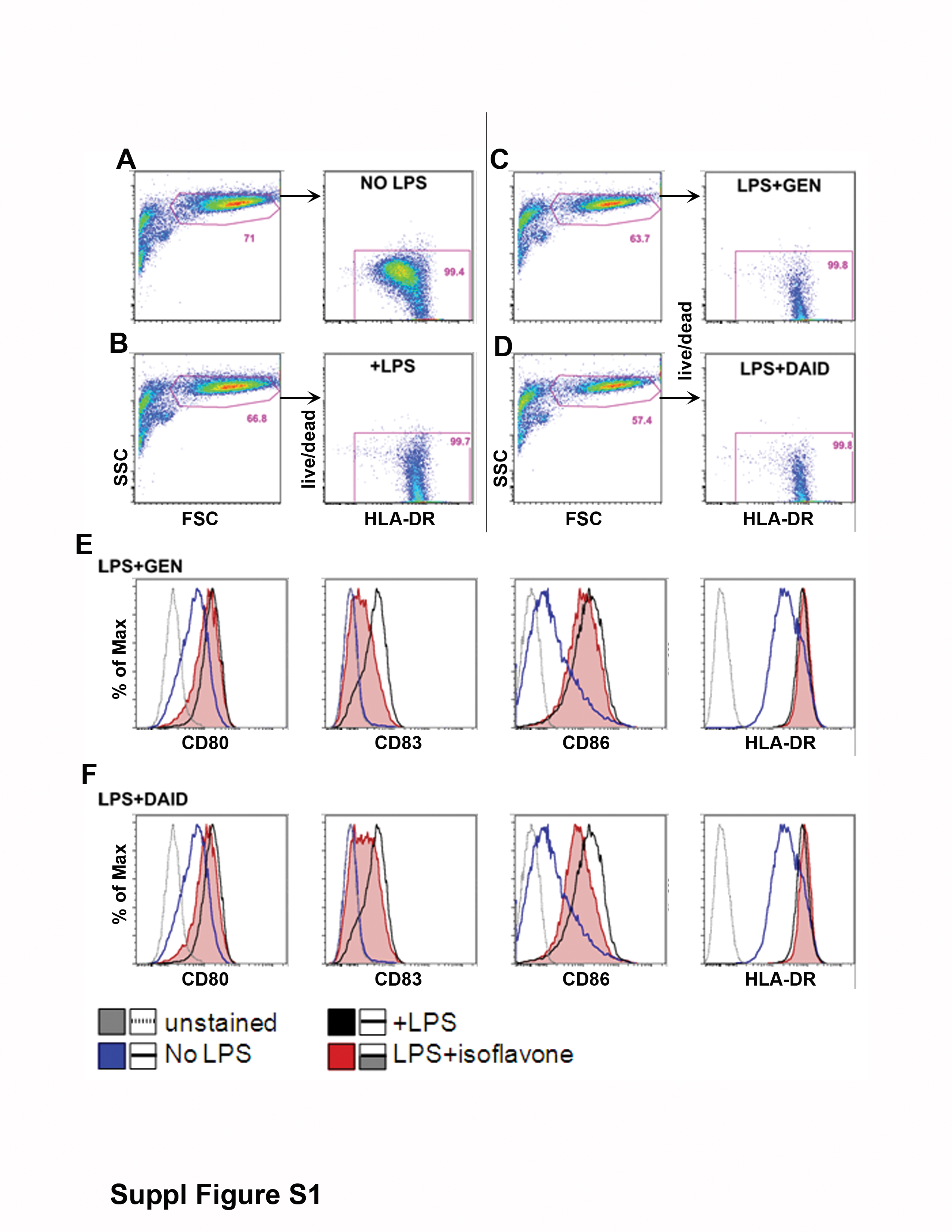

Supplement: Figure S1 — MDDC flow cytometry. The expression levels of CD80, CD83, CD86 and HLA-DR were tested in FSChi “live” MDDCs treated with LPS+/−genistein or daidzein. A representative staining is shown as dot plots (A-D) and histogram overlays (E&F). (TIF) [file pone.0047979.s001.tif]

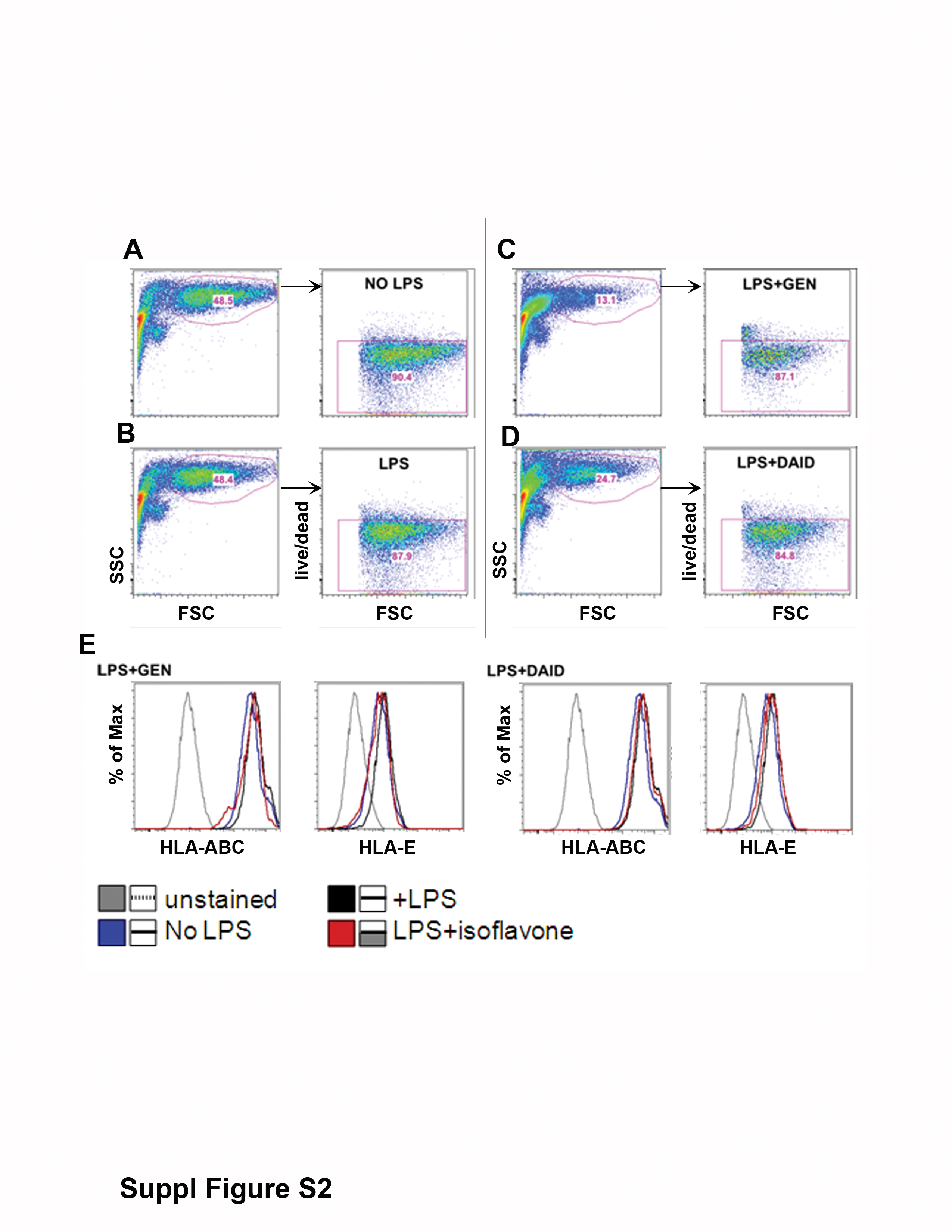

Supplement: Figure S2 — MDDC flow cytometry. The expression levels of HLA-ABC and HLA-E were tested in FSChi “live” MDDCs treated with LPS+/−genistein or daidzein. A representative staining is shown as dot plots (A-D) and histogram overlays (E). (TIF) [file pone.0047979.s002.tif]

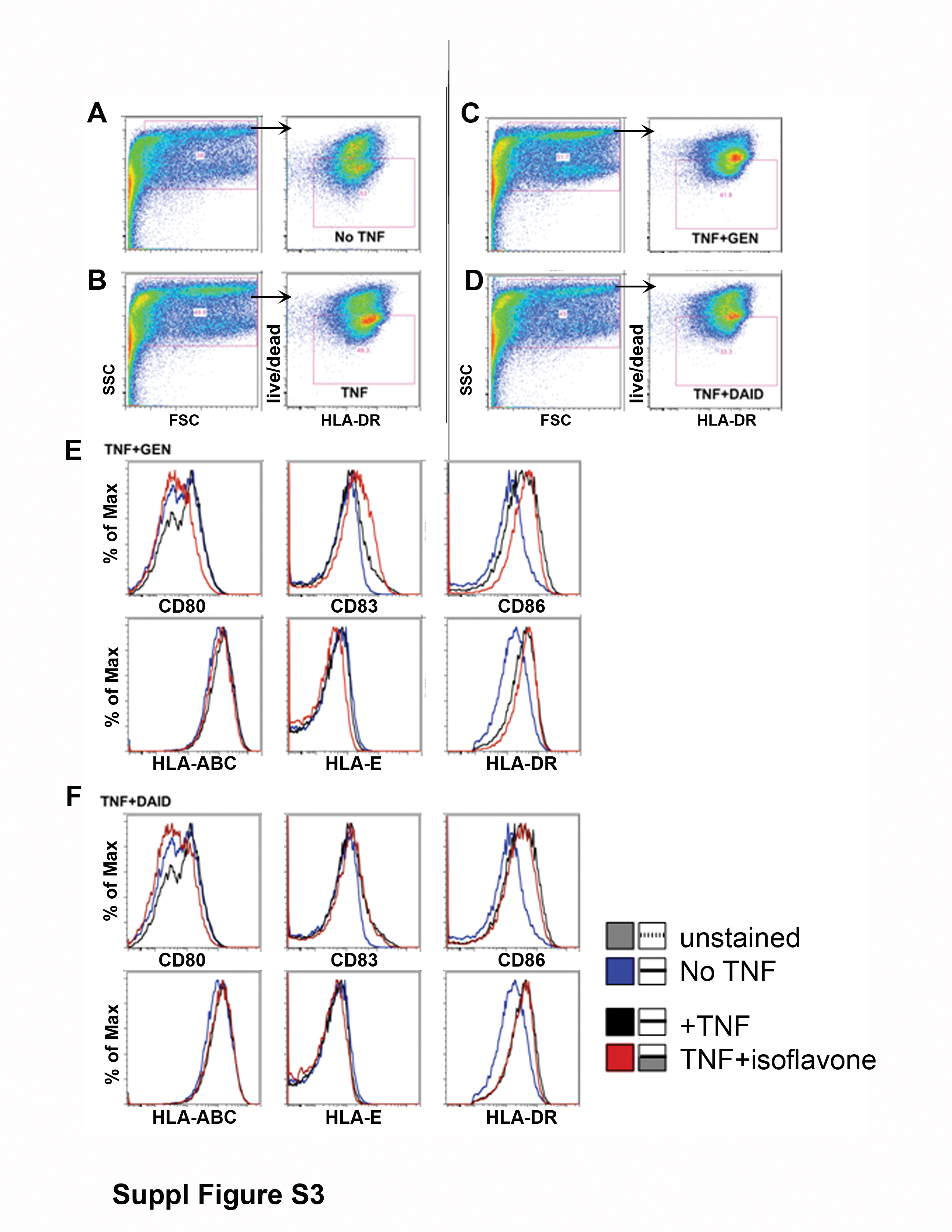

Supplement: Figure S3 — MDDC flow cytometry. The expression levels of CD80, CD83, CD86, HLA-ABC HLA-E and HLA-DR were tested in FSChi “live” MDDCs treated with LPS+/−genistein or daidzein. A representative staining is shown as dot plots (A-D) and histogram overlays (E&F). (TIF) [file pone.0047979.s003.tif]

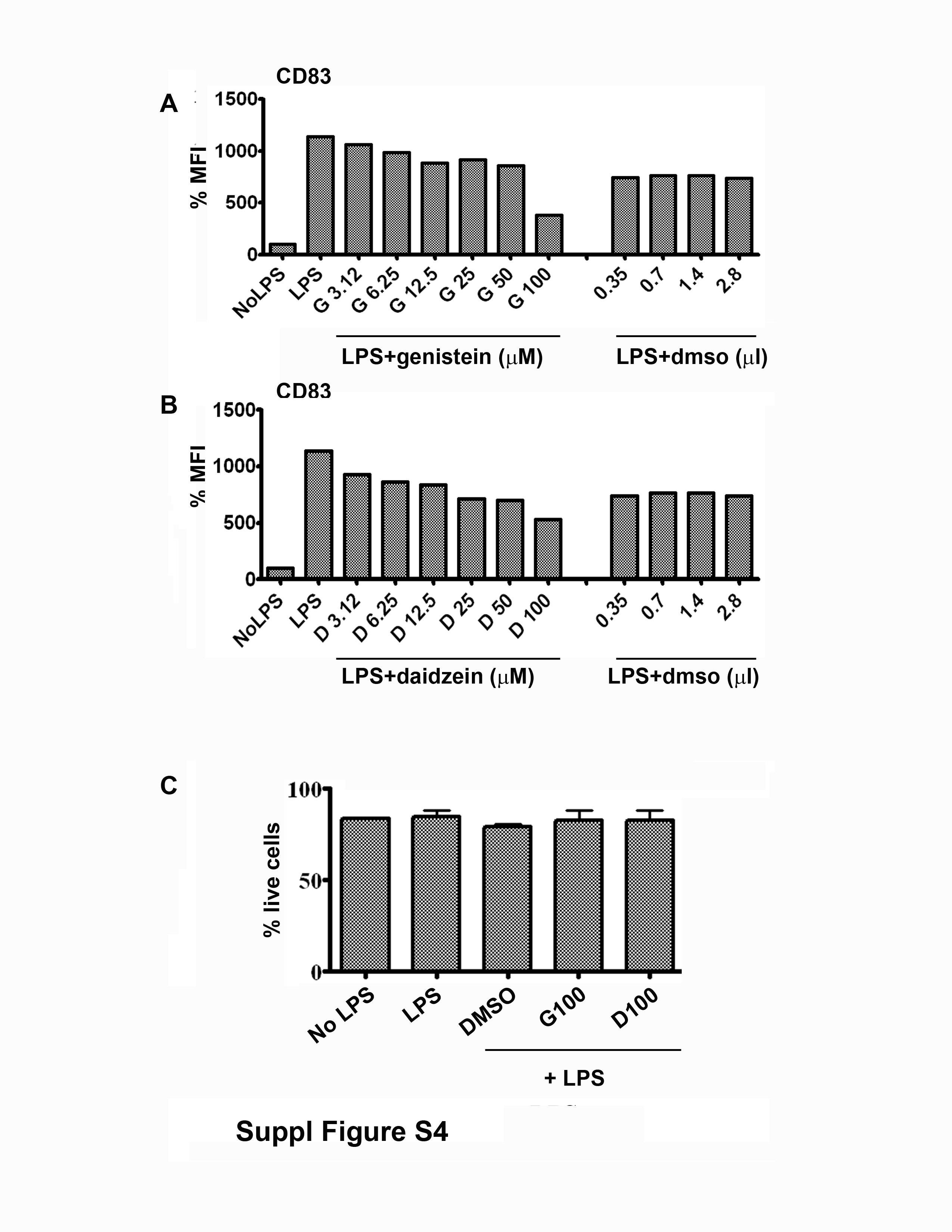

Supplement: Figure S4 — Isoflavone titration. MDDCs were activated with 100 ng/mL LPS in the presence or absence of titrated amounts of genistein (A) or daidzein (B). The concentration of isoflavones ranged from 3 µM to 100 µM. The percentage surface expression levels of CD83 were calculated from the geometric mean fluorescent intensities where unstimulated control (No LPS) was taken as 100% and shown as bar graphs. Vehicle control (DMSO, µL) for conditions from 12.5 to 100 µM are shown. Flow cytometric assessment of cell viability using live/dead stain (Invitrogen) was performed in cells treated LPS +/− isoflavones (100 µM) (C). No difference in cell death was found in all the conditions employed. (TIF) [file pone.0047979.s004.tif]

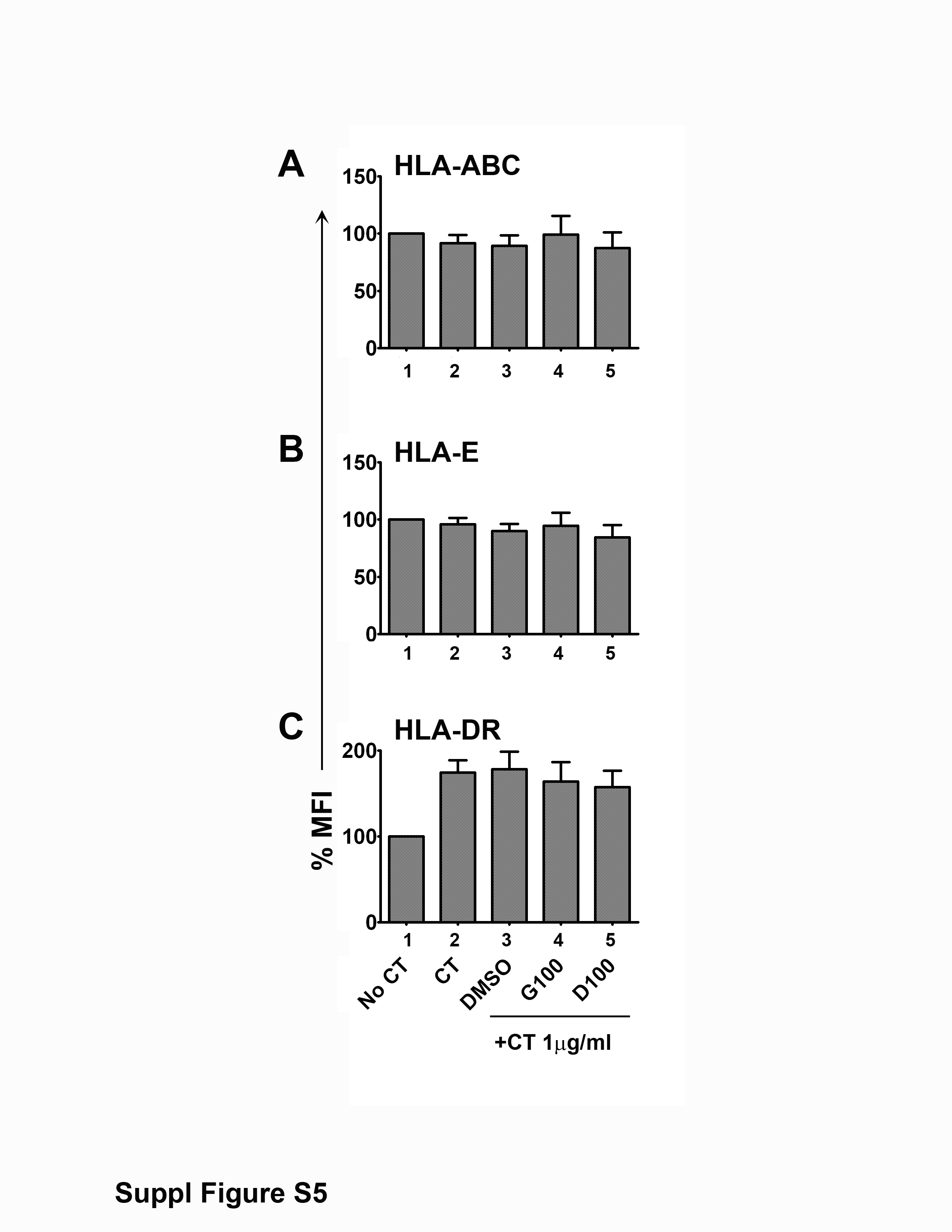

Supplement: Figure S5 — Effect of isoflavones on CT-induced MHC expression on DCs. MDDCs were activated with 1 µg/mL CT for 18 h and stained with HLA-ABC (A), HLA-E (B) and HLA-DR (C). DMSO - vehicle control for genistein and daidzein. The percentage surface expression levels were calculated from the geometric mean fluorescent intensities (No LPS or No TNF controls taken as 100%) and shown as bar graphs. The data pooled from at least 4 independent experiments from different cell donors. At least two replicates were performed for each condition in every experiment. There was no significance in the expression levels of these molecules between the conditions employed. (TIF) [file pone.0047979.s005.tif]

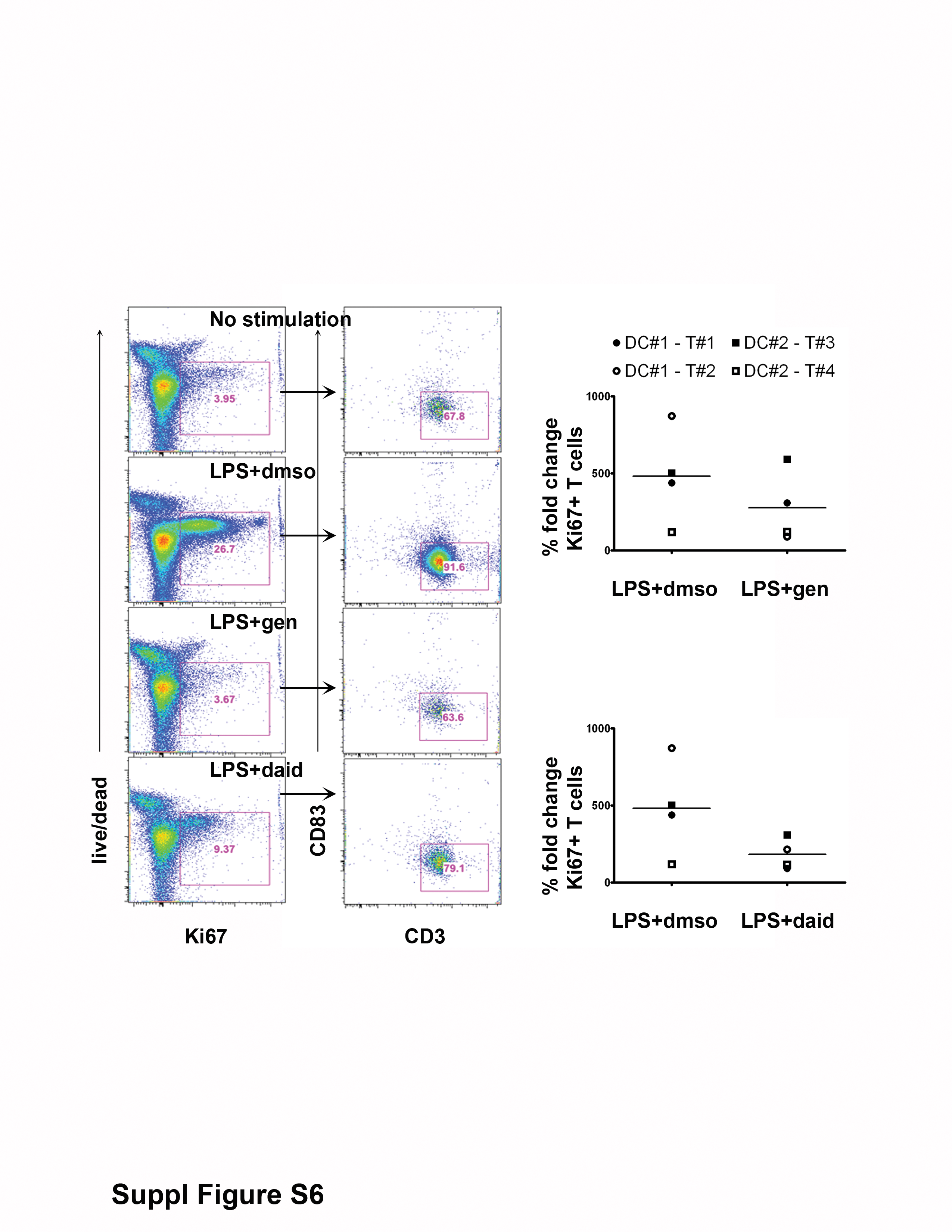

Supplement: Figure S6 — Isoflavones regulate DC-induced T cell proliferation. MDDCs from two donors were activated with 100 ng/ml LPS +/− 100 µM genistein (G100) or daidzein (D100) for 18 h and washed. Each donor DCs were incubated with two allogenic CD4+ naïve T cells from two other different donors for 5 days. The cells were stained with fluorescent-conjugated antibodies against CD3, CD83 and Ki67 along with live/dead discriminator dye. The frequency of CD3+CD83− cells in Ki67+ gate in No stimulation (No LPS) condition was taken as 100% and the fold change in Ki67+ T cells in LPS+dmso, LPS+genistein and LPS+daidzein cultures were plotted. (TIF) [file pone.0047979.s006.tif]
